# Photosensitization agents for fs laser writing in PDMS

**DOI:** 10.1038/s41598-022-05366-w

**Published:** 2022-01-31

**Authors:** Jean-Sebastien Boisvert, Antsar Hlil, Sebastien Loranger, Ali Riaz, Yannick Ledemi, Younes Messaddeq, Raman Kashyap

**Affiliations:** 1 Department of Engineering Physics, Fabulas Laboratory, École Polytechnique Montréal, 2900 Édouard-Montpetit, Montreal, QC H3T 1J4 Canada; 2 Centre d’Optique, Photonique et Laser, Université Laval, 2375 Rue de la Terrasse, Quebec, QC G1V 0A6 Canada; 3 Department of Electrical Engineering, PolyGrames, École Polytechnique Montréal, 2900 Édouard-Montpetit, Montreal, QC H3T 1J4 Canada; 4 Département de Chimie, Faculté des Sciences et de Génie Pavillon Alexandre-Vachon, Université Laval, 1045, Avenue de la Médecine, Quebec, QC G1V 0A6 Canada

**Keywords:** Polymers, Materials for devices, Materials for optics

## Abstract

This study aims at identifying compounds incorporated into Polydimethylsiloxane (PDMS) which produce large refractive index change under fs laser exposition, potentially leading to optimal writing of waveguides or photonic devices in such a soft host. Germanium derivative, titania and zirconite derivatives, benzophenone (Bp), irgacure-184/500/1173 and 2959 are investigated. We show a mapping of the RI index change relative to the writing speed (1 to 40 mm/s), the repetition rate (606 to 101 kHz) and the number of passes (1 to 8) from which we establish quantitative parameters to allow the comparison between samples. We show that the organic materials, especially irgacure-184 and benzophenone yield a significantly higher maximum refractive index change in the order of 10^−2^. We also show that the strongest photosensitivity is achieved with a mixture of organic/organo-metallic material of Bp + Ge. We report a synergetic effect on photosensitivity of this novel mixture.

## Introduction

The Polydimethylsiloxane (PDMS) is a versatile material due to its biocompatibility, inertness, durability, elasticity, transparency, low cost, wide availability and ease of manufacture. This material has proved its importance in many fields such as microfluidics by facilitating the access to cheap and reproductible microfluidic chips^1^, in microfabrication where it is used as a photopatterning agent^2^ or in ultra-tunable photonics by acting as a host material for nanoparticle and gain medium to produce a widely tunable random lasing system^3^. Recently, the idea of using PDMS as a platform for femtosecond (fs) laser writing of waveguides has generated much interest amongst researchers because of its wide stretchability^4–6^. It can undergo a strain of 40% in the linear elastic regime and up to 170% before failure for bulk samples^7^. Laser writing on PDMS has been previously demonstrated using UV light^8^. A refractive index (RI) change (Δn) of ~ 0.14 has been reported but bleaching reaction of the PDMS was also observed resulting in degradation of the polymers chains which affects the mechanical and optical properties. The ideal goal is to render the PDMS photosensitive without significantly jeopardizing the other excellent qualities of this material, thus creating a cheap versatile writing platform for integration of photonics devices which would pave the way to ultra-tunable compact optical sensors, tunable lasers, or even tunable photonic crystals.

We have recently developed strategies to render PDMS photosensitive for fs writing^5,6^ by simply mixing the photosensitive agent, allytriethylgermane (a germanium derivate), into it before curing without altering significantly the optical and mechanical properties. A positive RI changes of Δn ≈ 0.004 was induced with fs laser exposure and writing of waveguiding structures, along with a diffraction grating, were demonstrated^9^, supporting the viability of integrating tunable photonics structures inside this new material. Panusa et al.^4^ also reported loading PDMS with phenylacetylene as a photosensitizing agent by diffusion, measuring a positive refractive index change of Δn ~ 0.06 and demonstrated light guidance after waveguide inscription. However, the process used is complex and takes several steps to achieve photosensitization of PDMS, which makes production relatively time consuming. Also, the photosensitivity may be short lived as the phenylacetylene solution has been reported to degrade over time when exposed to ambient light^10^.

**Table 1:** Potential photosensitizing materials under investigation.

Name	Commercial name	Nature	Chemical structure
Polydimethylsiloxane	PDMS	–	
Germaniun-ATEG	Allytriethylgermane	Organo-metallic	
Germanium-acrylate (MACMTG)	Methacryloxymethyltrimethylgermane	Organo-metallic	
Titanium oxide	NP-TiO_2_	Inorganic	
Zirconite	Zirconium (IV) isopropoxide	Organo-metallic	
Benzophenone (Bp)	Benzophenone	Organic	
IRGACURE-184	Hydroxcyclohexyl phenyl ketone	Organic	
IRGACURE-1173	Hydroxy-2 methylpropiophenone	Organic	
IRGACURE-2959	2-Hydroxy-4(2-hydroxyethyoxy)-2-methylpropiophenone	Organic	
IRGACURE-500	Hydroxcyclohexyl phenyl ketone-50% Benzophenone 50%	Organic	

Even though our previous results were promising^5,6^, still many questions need to be answered. The ideal candidate should produce a RI change, Δn of ~ 0.01 without significantly jeopardizing the other attributes of the polymer and remain stable over time. To achieve this goal, identification of compounds responsible for the production of the desired RI change under fs laser exposition is needed. This study focuses on investigating the effect of several potential doping candidates and writing parameters on the refractive index change.

**Figure 1:**
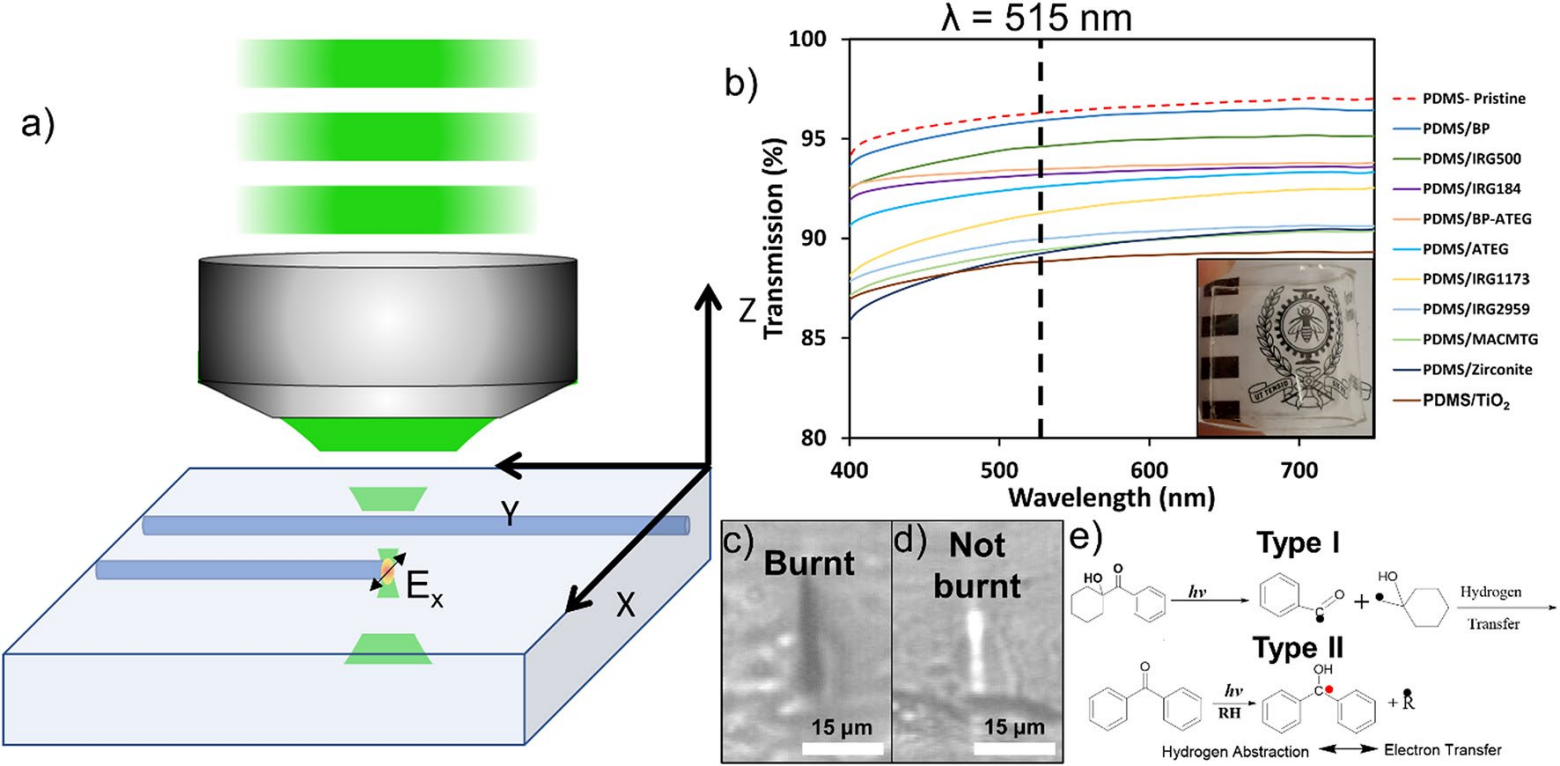
(**a**) PDMS fs laser writing scheme where the sample is moved relative to the focal spot to realize the inscription. (**b**) Transmission measurement of 1 mm thick samples. The inset shows a typical sample of photosensitized PDMS, (Irgacure-184) demonstrating that the PDMS is still flexible, transparent and without void clusters rendering it suitable for fs laser inscription. (**c**) and (**d**) display a near-field optical micrograph of the end-face cross-section of a burnt and unburnt waveguide respectively. In (**d**) we can clearly see that the 1 cm length waveguide is guiding white light. In (**e**) you have the generalization of the two photosensitive mechanism family, cleavage and hydrogen-abstraction.

The ideal photosensitive agents should produce free radicals efficiently, essential for the chemical modification resulting in the RI change and be transparent to the writing wavelength by staying in the multiphoton absorption regime. Furthermore, the size of the molecule, cost, odor, shelf life, solubility and color were important criteria in selecting those photosensitive agents to achieve samples which are well-blended, odorless, colorless, low cost, and without the presence of clusters. Reactivity of the processes is also a concern, since the energy is deposited and dissipated quite quickly, therefore reactive processes were more favored. The following mechanisms are known in photosensitive agents and may be the reason for the observed RI change. We used two types and combinations of photosensitive agents. Type I, is Cleavage mechanism photosensitive agent, (Fig. 1e) where the molecule is cleaved into two active radicals hunting for any other radicals in the system to react with. Type II, is the hydrogen-abstraction family photosensitive agent. After abstracting a hydrogen from the polymer chain forming two bounded radicals initiating a chemical reaction (Fig. 1e) where a hydrogen atom is transferred between the two involved species and which usually requires less activation energy than type I mechanism. In Table 1, all the photosensitive agents are type I except the benzophenone BP. These physical mechanisms may be the probable cause for the RI change, although further study, beyond the scope of this article, is needed to understand the reasons more fully. Once promising candidates are identified, the origins of the RI, the thermal stability, the optical quality, the chemical stability, and the stability of the RI change over time, can then be focused on. These aspects will be addressed in a forthcoming publication.

## Materials and methods

### Material

Dow SYLGARD™ 184 Silicone Elastomer was formulated from a base agent and curing agent, both of which were supplied by Ellsworth, while benzophenone (ReagentPlus® 99%), (1-hydroxycyclohexyl)(phenyl)methanone (Irgacure-184), (Irgacure-500) Hydroxcyclohexyl phenyl ketone-50% Benzophenone 50%, 2-hydroxy-2-methyl-1-phenylpropan-1-one (Irgacure-1173) and 2-hydroxy-1-(4-(2-hydroxyethoxy)phenyl)-2-methylpropan-1-one (Irgacure-2959), Zirconium (IV) isopropoxide, Xylene were purchased from Sigma-Aldrich. Allyltriethylgermane (ATEG) and Methacryloxymethyltrimethylgermane Germanium-Acrylate (MACMTG) were supplied by Gelest and were used as received.

### Methods

Sylgard-184, as our host PDMS which is a two-part polymer with a mixing proportion of 10:1 of base (part A) and curing agent (part B), was used for our investigations. The photosensitizing agent was incorporated into part B of the compound before mixing with part A. Then, the mixture was put into a sonicator for 10 min to ensure homogeneity of the final mixture. The still liquid PDMS was then poured into a mold and placed in a degasification chamber for 30 min to remove the air bubbles trapped by the previous mixing process. The bubble free mixture was then cured in an oven at 70 °C for one hour. Finally, the polymerized PDMS containing the photosensitizing agent was removed from the mold and cooled to room temperature before being used for fs laser writing. A slightly modified procedure for the preparation of Irgacure-2959 sample was used since direct incorporation of the powder into PDMS produces a whitish film, which is unsuitable for laser writing. A few drops of boric acid H_3_BO_3_ were added to the mixture to maintain acidity towards the end of the reaction without causing significant esterification. Before writing, a rough preselection of the samples was made based on the visual quality of their transparency as shown in Fig. 1b. Samples showing the presence of clusters or crystallization or signs of degraded mechanical quality (brittleness) were rejected. The samples were roughly tested by hand after writing and RI measurement to ensure they were still stretchable. Transmission spectrum of 1 mm thick sample were measured with the Varian Cary 60 spectrophotometer system. In Fig. 1b for Benzophenone, Iragacure184/500, the mixture of BP-ATEG and ATEG, the relative differences with the pristine PDMS sample is under 5% for all the visible spectra. A larger difference, in between 5 and 10%, was found with the other compounds (Irgacure1173/2959, Zirconite, TiO_2_ and MACMTG). Higher Rayleigh scattering, higher Fresnel reflection at the interfaces caused by higher RI samples coupled with the instrument error could be responsible for the difference.

Based on our previous experiments^9^, 250 fs laser pulses at a wavelength of 515 nm were used. Therefore, the absorption of two photons is needed to bridge the bandgap of the PDMS. The laser used for the writing was a Pharos (1030 nm, 250 fs) combined with an OPA Orpheus to get the desired 515 nm writing wavelength. The linear polarization (*E*_*x*_) was perpendicular to the writing direction as shown on Fig. 1a. The repetition rate of the laser was tuned from 101 to 606 kHz with a pulse picker to ensure that the pulse energy was conserved. The speed of writing was selected to be between 1 and 40 mm/s. These parameter ranges were used on every sample to extract a mapping of the RI change. The pulse energy was maintained at 6.6 nJ and measured after the writing lens which was an Olympus microscope objective with a numerical aperture (*NA*) of 0.4. The writing scheme is presented in Fig. 1a. A multi-pass strategy was used to enhance the exposition dose without submitting the sample to a single high energy pulse which would result in degradation of the PDMS that is termed as “burning” or being “burnt”, for simplicity. The difference between a burnt event and an unburnt event is shown in Fig. 1c,d, which are end-face near-field micrographs of samples exposed to fs laser exposure. Inscription has been performed 400 μm into the bulk of the PDMS samples. The measurement of refractive index change was performed using a RI measurement system the Ripper, on loan from Photonova Inc., which is an interferometric imaging system providing a tomographic phase image of the sample. Using this setup, we were able to extract the phase change between the unexposed materials and the exposed area and calculate the maximum phase change. Provided the height of the affected area is known from the waveguide’s dimensions^11^, (which is obtained by simple end-face cross-section imaging of the waveguide, as shown in Fig. 1c,d, the refractive index change can be calculated simply using Eq. (1).

1 $$\Delta n = \frac{\Delta \Phi \lambda }{{2\pi h}}$$

where *Δn* is the refractive index change, *ΔΦ* the phase change, *λ* the measurement wavelength and *h* the depth of the affected area. Based on the data provided by PhotoNova Inc., the system has a phase noise of 10 mrad which is equivalent to an RI change of 6 × 10^−5^ in our measurements.

The change in photosensitivity is assessed by comparing the samples with the intrinsic photosensitivity of the pristine PDMS sample. To facilitate the comparison between the samples, observations were based on a set of two factors. The first one is the maximum RI change induced in the sample. However, this parameter alone does not do justice to each dopant, as the RI change is highly non-linear (fast increase) before a burning event which renders a waveguide useless. This causes low reproducibility to attain the highest RI change as the risk of burning is high. Furthermore, this parameter is also susceptible to the specific selected writing parameters since it does not consider the probability that a higher RI change could be induced outside of the chosen parameters. Therefore, to mitigate this aspect, we changed our parameters (speed, number of passes & repetition rate) over a large range. These parameters were the same for every sample to allow us to make suitable comparisons.

The second factor is designed to bridge the uncertainty of a simple maximum. A criterion was defined which is a relative integrated “volume” of refractive index change over all the scanned parameters. This corresponds to a weighted sum (weight is the relative RI change) of a working sample (unburnt) over the total number of samples as shown in the following equation named the Weighted Writing Area (WWA):

2 $$WWA = \frac{{\sum {\Delta n_{i} } }}{{\Delta n_{\max } x_{Total} }}$$

where *Δn*_*i*_ is the measured RI change (a burnt event is treated as an undetected event here and given the value 0) for the *i*th recipe, *Δn*_*max*_ the maximum RI change of the distribution and *x*_*Total*_ is the number of recipes used. This criterion gives a measure of the reproducibility of an RI change established on a scale from 0 (no RI change) to 1 (all the same RI change). A small value indicates a RI variation which occurs only for a few values of the parameters, while a large value indicates a low variability of RI increase as writing parameters are changed. Note that this criterion is not related to the amplitude of the RI variation; a sample with low RI increase can have a high WWA.

It can be useful to group the speed of writing and the repetition rate by introducing a pulse overlap function to the fluence parameter presented in Eq. (3)^12^.

3 $$F\left( {\frac{j}{{m^{2} }}} \right) = \left( {1 - \frac{v}{{2R_{r} \omega_{0} }}} \right)\frac{{E_{1p} }}{{z_{r} \omega_{0} }}$$

where *F* is the fluence in J/cm^2^, *R*_*r*_ the repetition rate, *E*_*1p*_ is the energy of one pulse, *v* is the speed of writing, *z*_*r*_ the Rayleigh length and *ω*_*0*_ is the beam waist at the focal spot. This parameter gives an indication of the energy dose necessary to produce the observed RI change, offering a more general view of the evolution of the RI since several combinations of writing speed, repetition rate, pulse energy and focusing lens numerical aperture led to the same fluence. Although this generalization can suffer from imprecision since it does not consider the reaction of the material under exposition (thermal accumulation and diffusion, for example), it is a metric that does assist in covering many of the variables related to the laser exposure.

## Results

**Figure 2:**
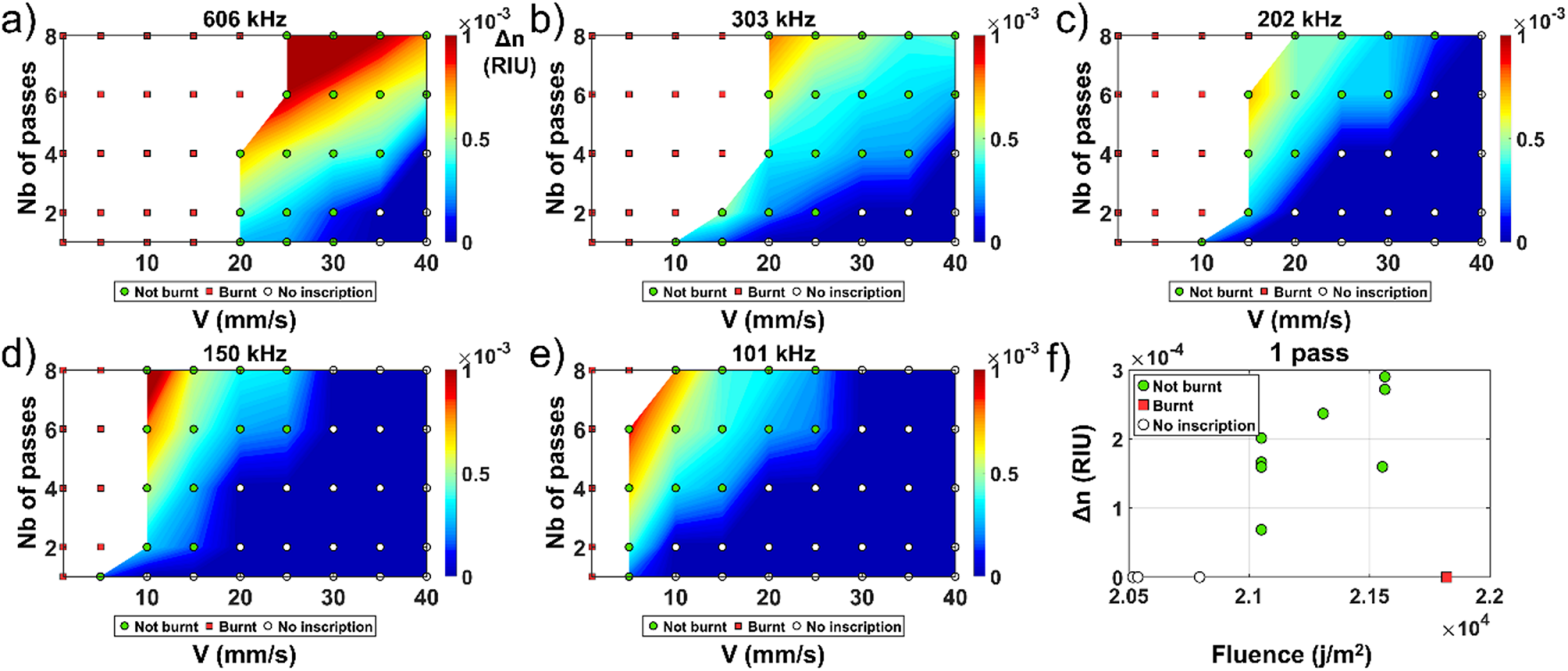
(**a**–**f**) Fs laser induced refractive index change in PDMS Sylgard-184 for various writing speed and numbers of passes at a given repetition rate. (**f**) Refractive index change evolution relatively to the fluence for one pass by varying the writing speed and/or the repetition rate incrementally.

The characterization covered by the chosen writing parameters applied to each of our sample is shown in the collection of 6 graphs presented in Fig. 2 for pristine Sylgard PDMS. This measurement set is used as a reference and gives insight on writing into un-photosensitized PDMS. Detailed results for each sample are available in the supplementary material section. A summary of the calculated factors is presented in Table 2 followed by a discussion on the refractive index change behavior observed for the best photosensitizing agents.

### Pristine Sylgard-184 polydimethylsiloxane (PDMS)

Writing into pristine PDMS was performed as a reference to evaluate the doped PDMS. It has been reported that the RI of this material will change according to the curing temperature used thus giving insight on the possible origin of RI change induced. The RI change seems to follow a saturation trend where the increase in refractive index decreases as the temperature increases^13^. The pristine PDMS used in this study was Sylgard-184 with a proper mixing ratio of the resin to curing agent of 10 to 1. The measured refractive index change induced by laser writing with respect to all writing parameters is shown in Fig. 2.

Writing in PDMS without a photosensitization agent is achievable as shown in Fig. 2. A maximum RI change of 1.3 × 10^−3^ was induced after 8 passes. However, many inscriptions resulted in burnt events especially at higher repetition rate (606, 303 and 202 kHz). Also, most of these inscriptions are in the order 10^−4^ which is 2 orders of magnitude below the desired target of 10^−2^ RIU. For single pass inscription, the writing parameters are confined to a well-defined set as shown in the fluence graph Fig. 2f. The RI evolution according to the writing speed seems to evolve differently at higher repetition rate (606, 303 and 202 kHz) compared to lower rate (˂ 150 kHz). The evolution at higher repetition rate seems to follow a linear or slightly quadratic behavior contrary to the behavior at lower repetition rate, which is more drastic, suggesting that heat accumulation could be a mechanism at play in the RI change. It can be noted that the index change with number of passes is relatively linear, which makes the tuning of the recipe easier. This also indicates each pass is not affected by the previous pass. For a single pass inscription, the maximum refractive index change induced was only 3 × 10^−4^. The RI change can be caused by a combination of densification of the material, breaking of the polymer chains or introduction of electronic defects, which will be reported on in a future study. However, the WWA is relatively high (0.114) which indicates that such recipes are easier to reproduce and work with for a wide set of parameters.

### Ranking of photosensitizing agent

In Table 2, the quantitative summary regarding the maximum RI change achieved and the score of the WWA parameters for all the photosensitizing agents are compiled. The purpose of this study was to identify the combination that photosensitizes PDMS and to rank them in terms of efficiency of the photosensitization. Our goal is to obtain photosensitization of PDMS without significantly jeopardizing the other essential properties of it (transparency, biocompatibility, stretchability), to create a new biocompatible/cheap writing platform featuring ultra-high stretchability. We compare those results with the reference pristine PDMS at the top of Table 2.

**Table 2:** Quantitative summary for each photosensitizing agent listing the maximum RI change and the weighted writing area.

Compound	Maximum *Δn* (RIU)	WWA
Polydimethylsiloxane (Sylgard-184)	0.001	0.12
Irgacure-184	**0.015**	0.07
Benzophenone	0.011	**0.15**
Irgacure-500	0.009	0.12
Irgacure-1173	0.003	0.08
Irgacure-2959	0.003	0.11
Titanium oxide	0.003	0.01
Germanium ATEG	0.004	0.03
Germanium acrylate (MACMTG)	0.002	0.02
Zirconium isopropoxide	0.004	0.04
Benzophenone and germanium-ATEG	**0.015**	0.10

Significant values are in [bold].

From Table 2, we can observe that all agents resulted in higher RI change than the pristine PDMS sample. However, the WWA parameters indicate that several of agents did not produce a significant RI change, or appreciable repeatability (WWA) compared to pristine PDMS. For organic materials, we can identify two candidates that reach a RI change in the order of 10^−2^, the Irgacue-184 with 1.51 × 10^−2^ and the Benzophenone with 1.07 × 10^−2^, for a respective mass concentration of 4% and 3%. Although the Irgacure-184 yields a higher RI change, its WWA value is half that of Benzophenone, which indicates that the recipe is more specific to some parameters and therefore may result in less reproducibility. Therefore, this material is more shielded against local perturbation and thus less sensitive to small recipe variations. However, it is important to recall that organic dopants can suffer from potential stability issues in the longer term.

To estimate the error in the measurement and give an idea of the reliability of the RI change induced, 50 inscriptions, each separated by 100 μm covering a width of 5 mm of the chosen sample, with the same writing parameters (4 passes, 20 mm/s, 606 kHz, 4 mW) were realized in an Irgacure-500 sample. This recipe was chosen since it provides a large enough RI change, in the order of 10^–3^ RIU which is larger than the resolution of the Ripper system (6 × 10^–5^). The average of the 50 measurement was 0.0036 with an uncertainty ± 0.0005 defined at 2σ to obtain a confidence level of 95%. This represents a ± 13% variation which includes the instrument error as well as the inhomogeneity distribution of the photosensitive materials in the samples, laser fluctuation effects, or presence of dust of imperfection along the optical path length resulting in reduced power exposition at the focal spot. The details of the measurements are presented in supplementary materials.

## Discussion

Inorganic materials and organo-metals used as dopants are much less effective in rendering the PDMS photosensitive and yield very low WWA, indicating that generating high RI change may be difficult to reproduce as the conditions are very parameter specific. Their incorporation into PDMS was generally more difficult since aggregation was often found due to high concentration or due to unbinding to the polymer chains. Low RI changes was systematically found at lower fluence (101 kHz) compared to pristine PDMS which indicates that the mechanism(s) behind the RI change are less favorable. This could be the case if the RI change is in part heat driven where inclusion of such inorganic materials would increase the heat conductivity, leading to less heat accumulation because of the increased heat diffusion. Despite their poor performance, inorganic materials yield better long-term stability and their higher heat conductivity can be an advantage for heat management of a device.

To mitigate the disadvantages of both cases, a mixture of both compounds composed of 2% Bp and 2% Ge-ATEG was attempted. Surprisingly, the fs laser induced refractive index change was increased to 1.52 × 10^−2^. Despite half of the concentration of Bp alone, this compound did achieve higher result which could not be explained by the simple addition of the contribution of Ge-ATEG alone. Therefore, these two-compounds work synergistically when exposed to fs laser radiation to produce a higher RI change. The mechanism behind this is not yet understood and it is not clear at this moment if it comes from a chemical interaction between Bp and Ge, the enhanced capability of Ge to deal with heat or a combination of both. One can also observe that the WWA was slightly lower than for the benzophenone itself. Notwithstanding, the WWA is still significantly higher than the Irgacure-184, the other agent which produced a similar RI change. It can be surmised that this mixed compound might provide an improved long-term stability and more effective heat management than organic dopants alone.

Irgacure-500 is a commercially available liquid mixture of 50% benzophenone and 50% Irgacure-184 which is commercially used as an initiator of photopolymerization. Since, Irgacure-184 did produce the maximum refractive index change and benzophenone the highest WWA, this compound was investigated further. The maximum RI change induced was of 9 × 10^–3^ with a WWA of 0.12 for a successful incorporation of 4% wt. As one can observe in Table 2, this photosensitive agent scores lower than the benzophenone in both parameters and lower in the maximum RI change for Irgacure-184 but higher for the WWA. Detailed results are available in the supplementary material.

Irgacure-1173, also called Darocur and Irgacure-2959 are two well know commercial UV-photo-initiators. Darocur is a liquid compound which has been successfully incorporated into Sylgard PDMS. The performance of these materials was shown to be relatively poor compared to the other organic dopants. The maximum RI change was measured to be 3.2 × 10^−3^ and 2.5 × 10^−3^ for Irgacure-1173 and Irgacure-2959 respectively. Detailed results are available in the supplementary material.

Germanium, Zirconium, and Titania derivatives were also incorporated into PDMS. Photosensitization through germanium derivatives has already been demonstrated in our previous work in^9^ and a maximum RI change under different writing conditions (NA writing lens 0.25, 3.3 nJ) of 2.2 × 10^−4^ after 34 passes was reported. We have tested such samples with an extended range of parameter to compare them to our other dopant candidates. We could achieve a maximum RI for Germanium-ATEG of 3.6 × 10^−3^ and 1.4 × 10^−3^ for Germanium acrylate. Again, the WWA is quite low (0.027) compared to pristine PDMS. The detailed results are available in the supplementary material. Relative to the writing parameters used in this study and compared to other photosensitive agents, we demonstrated that the photosensitivity enhancement is small compared to other solutions shown in this paper. The same conclusion can be drawn for the Zirconium and Titania compounds where maximum RI induced were 3.7 × 10^−3^ and 2.8 × 10^−3^ with WWA of 0.037 and 0.005, respectively. For Zirconium, the initiator (Part A) of the Sylgard mixture was removed since its addition with Zirconium results in a whitish film, unsuitable for fs laser writing. Surprisingly, Zirconium acts on its own as an initiator enabling the polymerization process to occur and creates a transparent film. Therefore, we can establish 3 potential candidate that compose the top tier of our list, the Irgacure-184, the benzophenone and the mixture benzophenone-germanium. Closer examination of these candidates will be given in the following section. Use of those photosensitive agents in other types of PDMS is yet to be investigated to determine their versatility across different types of PDMS but is part of a complementary article. Preliminary results from ageing over a period of 6 weeks at room temperature (under ambient illumination) of these 3 candidates showed no significant change in the induced RI change. Detailed information will be given in a complementary article on the characterization of these novel candidates.

### Irgacure-184

**Figure 3:**
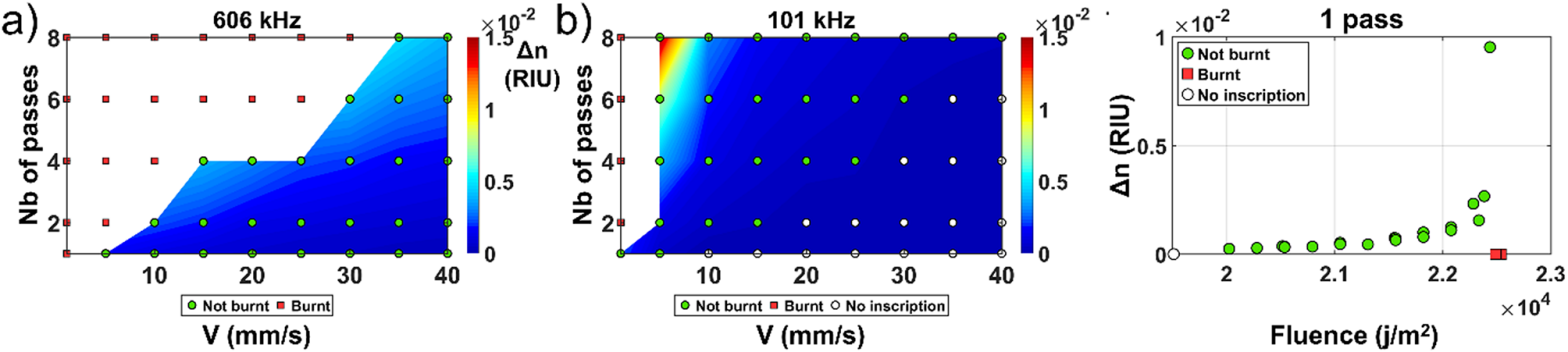
Fs laser induced refractive index change in PDMS-Irgacure-184 for various writing speed and numbers of passes at (**a**) 606 kHz and (**b**) 101 kHz. (**c**) Refractive index change evolution relatively to the fluence for one pass by varying the writing speed and/or the repetition rate incrementally.

Irgacure-184 is a commercial compound that can be simply incorporated into PDMS at the mixing stage just before curing. Prior to incorporation, this compound needs to be diluted in xylene to ensure proper mixing. It has been already used as a photo-initiator in the case of UV polymerization in a sol–gel material^14^. The concentration incorporated was 3% mass in the mixture. The measured refractive index change is shown in Fig. 3 for different repetition rates, writing speed and number of passes.

The maximum RI induced was 1.51 × 10^−2^ (101 kHz, 8 Passes, 5 mm/s) with a WWA of 0.07, which is 11 times higher than the maximum RI change induced in pristine PDMS, but more localized to specific parameters. RI changes in the order of the 10^−3^ were observed for every repetition rate contrary to what was observed in the pristine PDMS. Compared to pristine PDMS, the number of burnt events is lower which means that this compound is more robust with respect to the writing process. For a fixed repetition rate and number of passes, the RI change grows suddenly before burning occurs as the writing speed is reduced as we can observe on the sharpness of the color gradient on Fig. 3b. This phenomenon is less pronounced as we increase the repetition rate. This behavior is also well expressed in the fluence graph Fig. 3c. This seems to indicate that part of the observed RI change is thermally driven. As the first pulse arrives in the material, the material will suffer a drastic increase in temperature followed by an exponential decay until equilibrium, as shown in^15^. As we increase the thermal decay overlap between the pulse, the residual heat accumulates with the heat of the new pulse increasing the temperature. When the temperature exceeds 265 °C, the material degrades and burns^9^. We can observe that the measured RI does not significantly change for different combinations of writing speeds/repetition rates resulting in the same fluence value as is shown in Fig. 3c.

A careful examination of Fig. 3 shows that the dependence of RI change with respect to the number of passes is approximately quadratic. This is likely caused by the production of electronic defects such as color centers as reported^16,17^ where the previously inscribed defects increase absorption of light of the successive laser passes, thus increasing the subsequent RI change. This behavior is well expressed in Fig. 3b where this quadratic increase enables the transition from a RI change of 5 × 10^−4^ (1 pass) to 1.5 × 10^−2^ (8 passes) for a speed of writing of 5 mm/s. The fact that a similar RI change is observed at 10 mm/s, 2 passes, but without the strong quadratic expansion afterward reveals that fluence, rather than thermal accumulation plays a role in the nature of the RI modification. The exact nature of this RI change is still under investigation.

### Benzophenone

**Figure 4:**
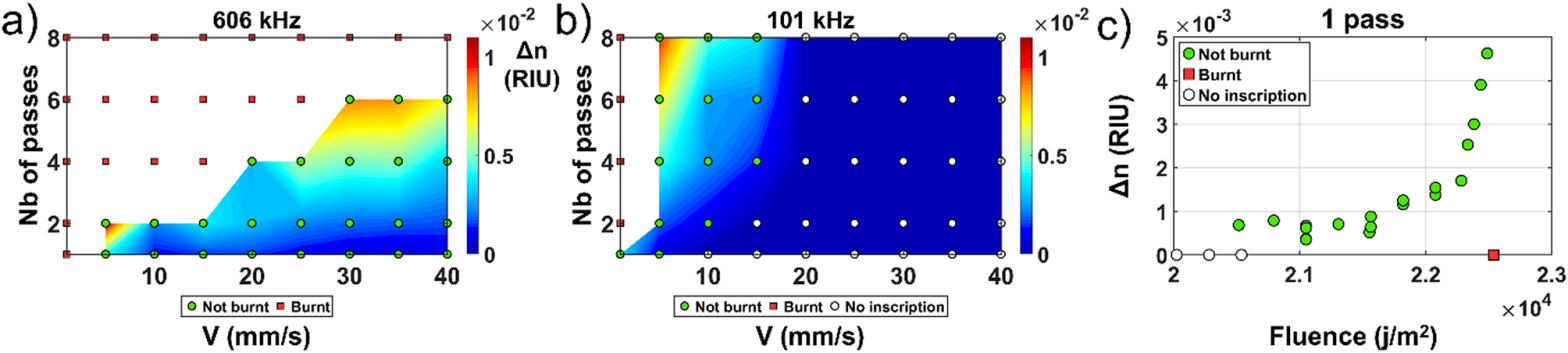
Fs laser induced refractive index change in PDMS-Bp for various writing speed and numbers of passes at (**a**) 606 kHz and (**b**) 101 kHz. (**c**) Refractive index change evolution relative to the fluence for one pass by varying the writing speed and/or the repetition rate incrementally.

Benzophenone (Bp) is a well know photo-initiator and has been reported as a potential photosensitizing agent in our previous work^6^. This commercial compound comes in a solid form and needs to be dissolved in xylene before incorporation in the PDMS. One must be careful including Bp into a PDMS host since excessive concentration of Bp leads to formation of undesirable aggregates in the PDMS mixture. Here, we report that a concentration of 4% of Bp was successfully integrated into PDMS without aggregation. The RI change with respect to our writing parameters is summarized in Fig. 4.

The maximum RI change achieved was in the order of the 1 × 10^−2^ which is about 10 times higher than pristine PDMS, with a slightly higher WWA of 0.146. For a single pass, the maximum achieved refractive index change was 4.3 × 10^−3^ which is 14 times higher than PDMS. In Fig. 4c, one can observe that the fluence writing margin is much higher for this compound (similar to the Irgacure-184) than PDMS and that the maximum fluence before observation of burnt events is higher. This indicates that the material is more suitable to deal with the fs laser exposition. Here again, the writing process has a threshold-like behavior as we increase the fluence (maximum value of fluence before burning). However, more variability in the RI measurement compared to the Irgacure-184 for the same fluence is found.

Careful examination at same fluence reveals systematically that higher RI changes are produced with a lower speed/repetition rate recipe. However, this variation is small compared to the general threshold behavior trend shown in Fig. 4c. We can hypothesize that once the light is absorbed by the material, there will be a different process at play (multiphoton ionization, energy transfer and thermodynamic processes) leading to diffusion of the generated heat out of the focal volume. Assuming that the repetition rate is faster than the thermal equilibrium time, which is the case for repetition rate higher than 100 kHz in glass^18^, a slower writing speed will result in more contribution from previous writing events providing an extra starting base resulting in a smaller increase in the RI. If this hypothesis is true, thermal annealing of this contribution to the RI should be observed and it will be evaluated in a future study.

### Benzophenone and germanium-ATEG

**Figure 5:**
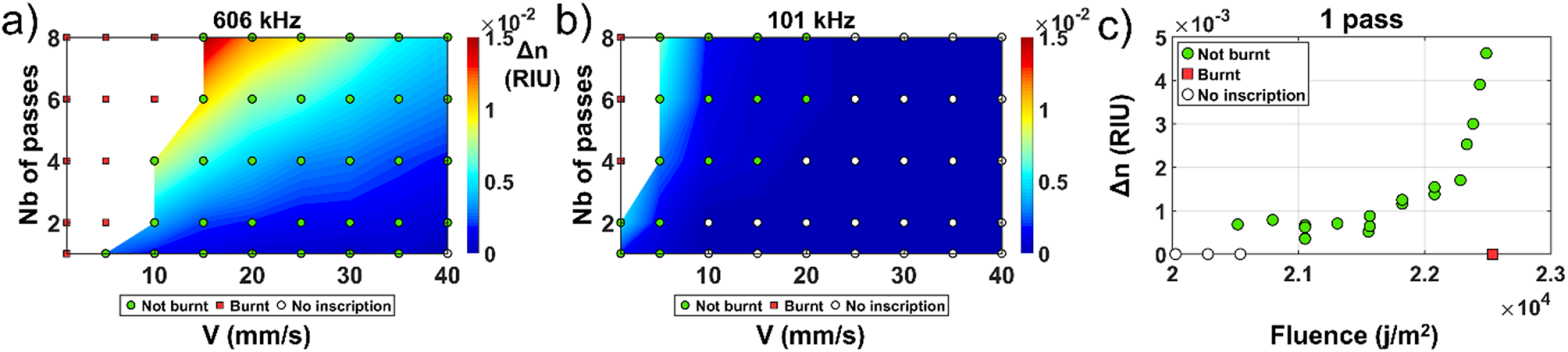
Fs laser induced refractive index change in PDMS-Bp-Ge for various writing speed and numbers of passes at (**a**) 606 kHz and (**b**) 101 kHz. (**c**) Refractive index change evolution relatively to the fluence for one pass by varying the writing speed and/or the repetition rate incrementally.

The poor performance in terms of RI change induced by inorganic materials, contrary to organic material, is an incentive to work with only organic materials. However, organic materials such as benzophenone are well known to degrade overtime which could be a concern if it is not well attached to the polymer chains. Furthermore, inorganic materials render the PDMS more able to deal with the heat deposition since they are better heat conductors as is shown in^9^ where germanium TEG increases the heat flow by 3%. Therefore, it was decided to mix organic with an organo-metallic material, in this case Bp and Ge-ATEG, as a 2–2% mass concentration into the PDMS. In Fig. 5 the RI induced change with the different writing parameters are presented.

The maximum measured RI change was 1.52 × 10^−2^ which is the highest RI change registered for all the tested materials. The WWA is 0.104 which is similar to pristine PDMS. Once again, as fluence increases, the behavior of RI change is exponential before it burns catastrophically. Careful examination of the plots in Fig. 5 shows a seemingly quadratic behavior of the RI change with number of passes, as was observed in the pure Bp graph. One can also observe that even with half the concentration of Bp and with the poor performance of Ge-ATEG by itself, the combination of those two compounds inside the PDMS contribute more than their individual parts. This suggests a synergy between these two compounds. Although we do not yet have any clear and detailed explanation for this, we believe it could be a chemical interaction between the two compounds and/or the fact that the new material is more capable of processing the heat deposited by laser writing. The process behind this RI change is still under investigation and will be subject of a complementary future report.

## Conclusion

In this study we investigated the ability of organic (Irgacure-184/500/1173/2959 and Benzophenone), inorganic (TiO_2_ and Zirconium) and organo-metallic (Ge-ATEG and Ge-acrylate) compounds incorporated into PDMS to produce RI changes under fs laser irradiation. We first presented as a reference measurement, the impact of fs laser writing on pristine Sylgard PDMS and produced a unique 3D color mapping of the RI change induced relative to the writing speed, the repetition rate and the number of passes. From these graphs, we established two quantitative parameters in the form of the maximum RI change and the weighted writing area (WWA) giving a measure of recipe stability and reproducibility. We found that the organic material worked better than the inorganic ones (higher RI change, higher WWA) and we were able to identify two promising compounds: Benzophenone and Irgacure-184. We also found that a mixture of organic with the organo-metallic, in this case Bp + Ge, showed interesting results and was found to be the best photosensitive compound with an acceptable WWA, amongst the samples studied. We were able to select 3 compositions, the BP, Irgacure-184 and Bp + Ge as the best photosensitizing candidates. Further study can help understand the mechanism behind the refractive index change to further optimize each recipe. Also, characterizing the chemical, mechanical and thermal stability of each of these best candidates would be helpful for future device engineering. Nevertheless, this study sets out the foundation for a cheap, manufacturable and highly stretchable fs writing platform which pave the way for fabrication of widely tunable as well as flexible photonic devices such as waveguide devices, diffraction gratings, Bragg gratings, photonics crystals, which are all critical part of photonics in sensing and other applications.

## Supplementary Information

Supplementary Information.
