# Supplementary material for: Photosensitization agents for fs laser writing in PDMS
Source: Sci Rep. 2022 Jan 31;12:1623. doi: 10.1038/s41598-022-05366-w (PMC8803880; doi:10.1038/s41598-022-05366-w)
Supplement: Supplementary file 1 — Supplementary Information. [file 41598_2022_5366_MOESM1_ESM.docx]

Supplementary material

Photosensitization agents for fs laser writing in PDMS

Jean-Sebastien Boisvert^1,*^, Antsar Hlil^2,3,4,*^, Sebastien Loranger^1^, Ali Riaz^4^, Yannick Ledemi^2^, Younes Messaddeq^2,4^ and Raman Kashyap^1,2,3^

^1^École Polytechnique Montréal, Department of Engineering Physics, Montréal, 2900 Édouard-Montpetit, QC, H3T 1J4, Canada
^2^Université Laval, Centre d’optique, Photonique et Laser, Québec, 2375 Rue de la Terrasse, QC, G1V 0A6, Canada
^3^École Polytechnique Montréal, Department of Electrical Engineering, PolyGrames, Montréal, 2900 Édouard-Montpetit, Qc, H3T 1J4, Canada
^4^Université Laval, Département de chimie, Faculté des sciences et de génie Pavillon Alexandre-Vachon, Québec, 1045, avenue de la Médecine, Qc, G1V 0A6, Canada
[^*^*jean-sebastien-2.boisvert@polymtl.ca*](mailto:*jean-sebastien-2.boisvert@polymtl.ca)*,* [**antsar.hlil@polymtl.ca*](mailto:*antsar.hlil@polymtl.ca)

Irgacure-184

Full data set of the refractive index change for a 3 % irgacure-184 PDMS compound.


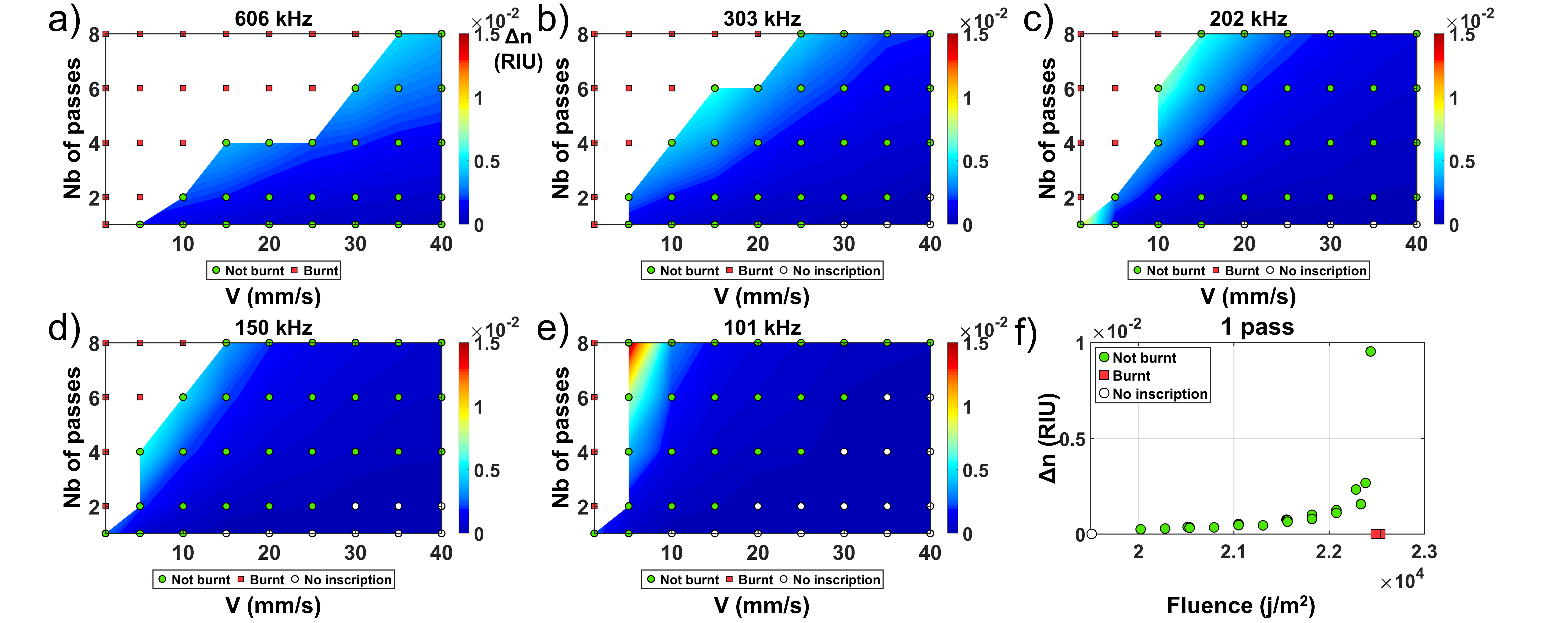


Supplementary Fig. S1: a), b), c), d) and e) Fs laser induced refractive index change in PDMS-Irgacure-184 for various writing speed and numbers of passes at a given repetition rate. f) Refractive index change evolution relatively to the fluence for one pass by varying incrementally the writing speed and/or the repetition rate.

Benzophenone

Full data set of the refractive index change for 4 % benzophenone PDMS compound.


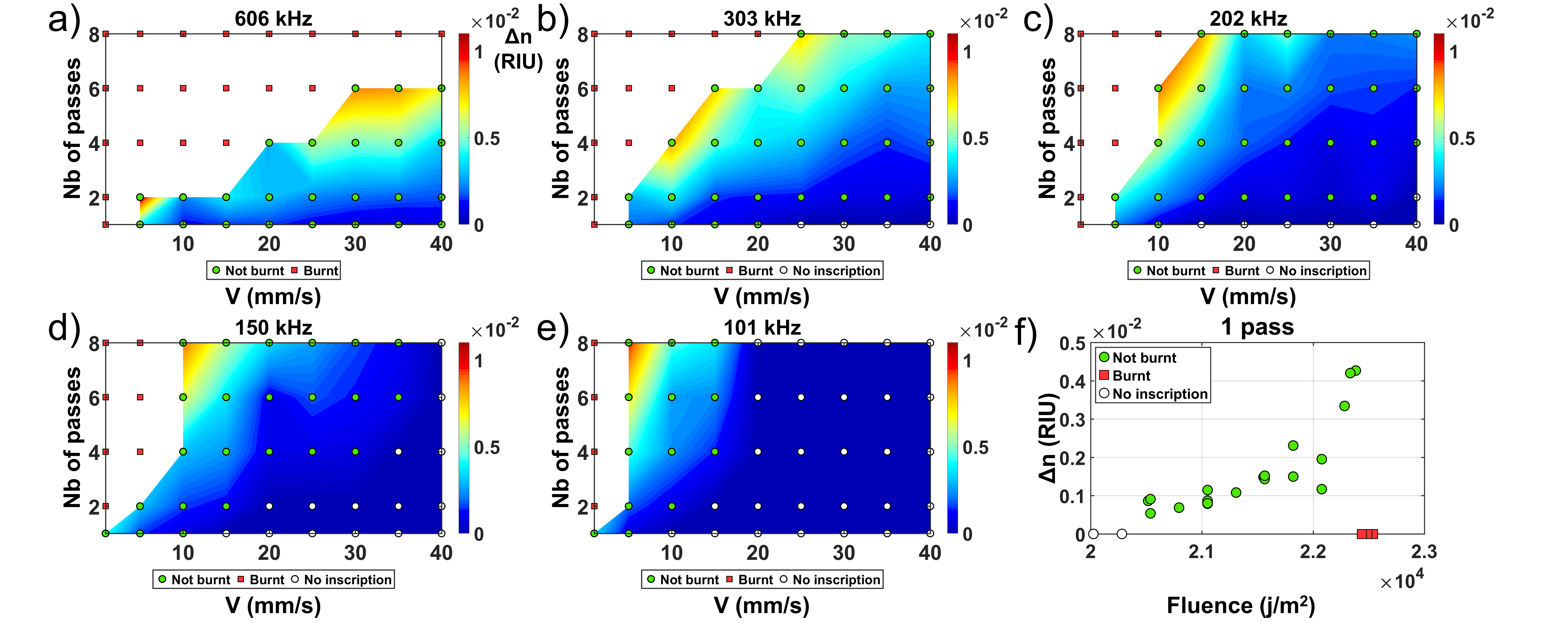


Supplementary Fig. S2: a), b), c), d) and e) Fs laser induced refractive index change in PDMS-benzophenone for various writing speed and numbers of passes at a given repetition rate. f) Refractive index change evolution relatively to the fluence for one pass by varying incrementally the writing speed and/or the repetition rate.

Benzophenone and Germanium-ATEG

Full data set of the refractive index change for 2 %-2 % Germanium ATEG-benzophenone PDMS compound.


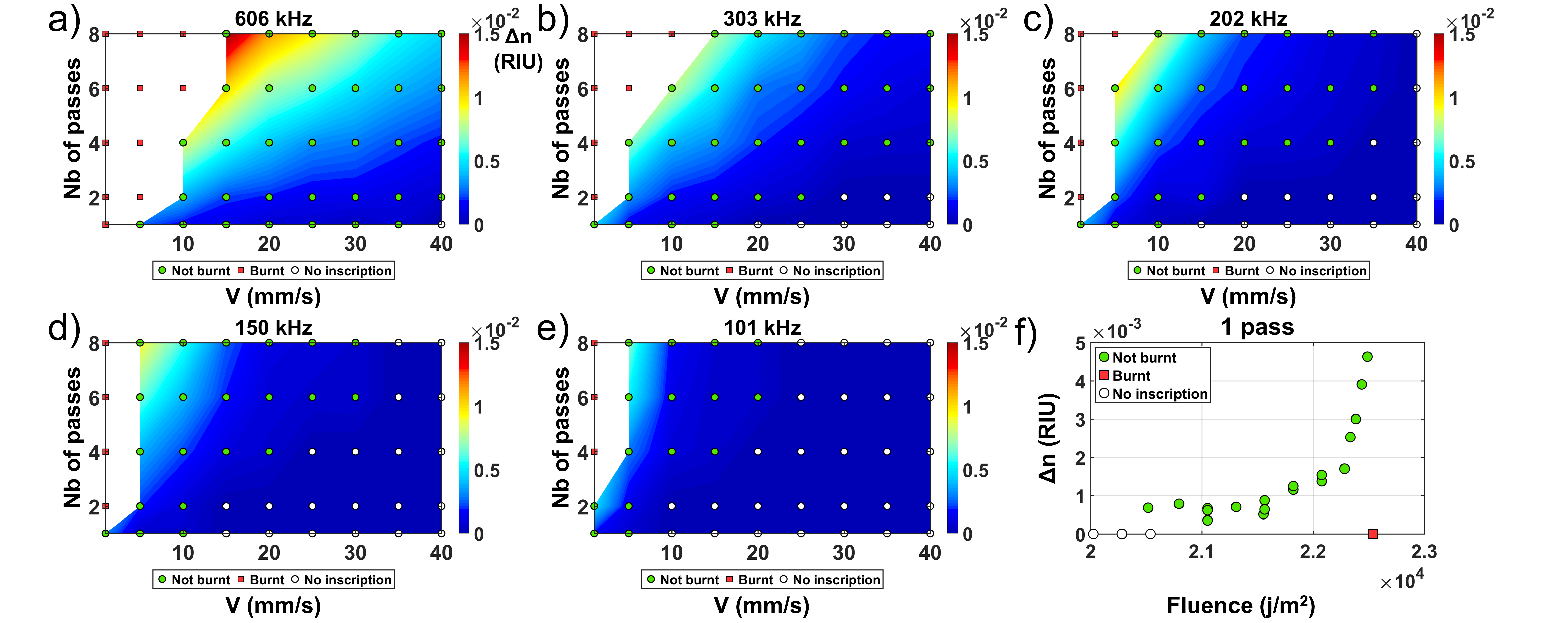


Supplementary Fig. S3: a), b), c), d) and e) Fs laser induced refractive index change in PDMS-Bp-Ge for various writing speed and numbers of passes at a given repetition rate. f) Refractive index change evolution relatively to the fluence for one pass by varying incrementally the writing speed and/or the repetition rate.

Irgacure-1173

Irgacue-1173 also know as Darocur, is a commercial UV-photoinitiator composed of 2-Hydroxyl-2-methyl-propiophenon which come in a liquid form. Concentration of 3 % mass was added at the mixing stage to the PDMS. The evolution of the RI for different composition of writing speed, repetition rate and number of passes is shown at Supplementary Fig. S4.


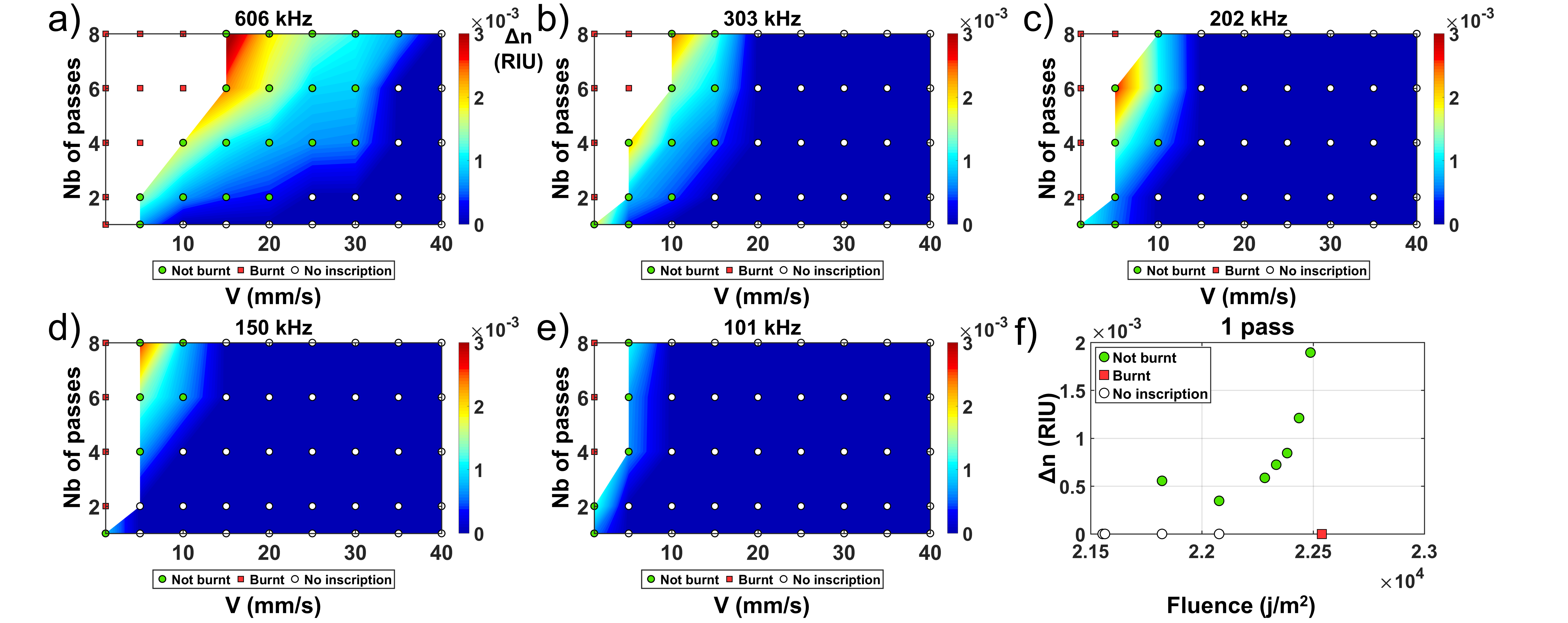


Supplementary Fig. S4: a), b), c), d) and e) Fs laser induced refractive index change in PDMS-Irgacure-1173 for various writing speed and numbers of passes at a given repetition rate. f) Refractive index change evolution relatively to the fluence for one pass by varying incrementally the writing speed and/or the repetition rate.

The maximum RI change measured was of 3.2 x 10^-3^ which is 3 times higher than the pristine PDMS. If we look at the fluence at one pass supplementary Fig. S4 (f), the behavior appears to be threshold-like and shifted at higher fluence value compared to the pristine PDMS. However, one can observe that there is two contradictory points at lower fluence value where we have one detected and undetected event for the same fluence. Once again, the two undetected events belong to the writing regime at higher speed. For a single pass inscription, the maximum refractive index achieve was still 6 times higher than the pristine PDMS. However, this difference seems to decrease with an increasing number of passages at higher repetition rate (606 kHz) where we found an increase of only 3 times of the maximum RI. Concerning, the relation between the number of passes and the RI change, the relation appears to be linear across all the graphs presented in supplementary Fig. S4.

Irgacure-2959

Irgacure-2959 is also a commercial UV photoinitiator which came in a solid form. Direct incorporation at the mixing stage of this compound result on a whiteish PDMS sample which is not suitable for laser writing. However, mixing it with a little bit (0.05 % wt.) of boric acid H_3_BO_3_remove this whiteish aspect and render the PDMS mix transparent again, therefore suitable for laser inscription. The RI change relatively to different recipe is presented at supplementary Fig. S5 for a 2 % wt. concentration.


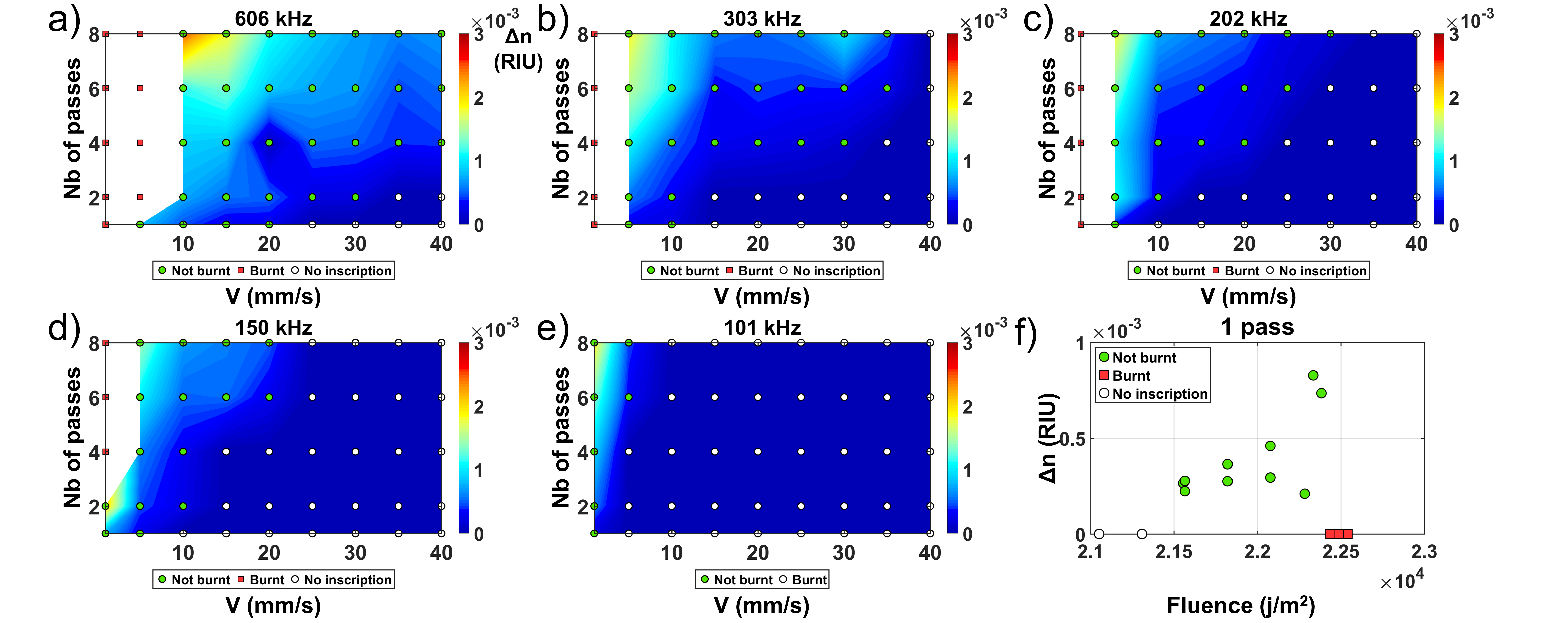


Supplementary Fig. S5: a), b), c), d) and e) Fs laser induced refractive index change in PDMS-Irgacure-2959 for various writing speed and numbers of passes at a given repetition rate. f) Refractive index change evolution relatively to the fluence for one pass by varying incrementally the writing speed and/or the repetition rate.

The maximum RI induced was of 2.5 x 10^-3^ which is still 2 time higher than pristine PDMS and is observed for every fixed repetition rate graph presented at supplementary Fig. S5. If we look across the graph of supplementary Fig. S5, there seems to be an asymptotical behavior with the fluence as we can also observe on supplementary Fig. S5 (f). The range of fluence where we observed inscription is slightly higher than the PDMS itself. However, for a single inscription both RI change is twice higher.

Germanium -ATEG

Germanium TEG have already been used as a photosensitive agent for PDMS in our previous work where we did register a maximum RI change of 3.6 x 10^-3^ for 34 passes using a lens of 0.25 of NA and a pulse energy of 3.3 nJ. Here, we show an extended version of this compound by showing the mapping of the RI change regarding a set of parameters as it is shown on supplementary Fig. S6.


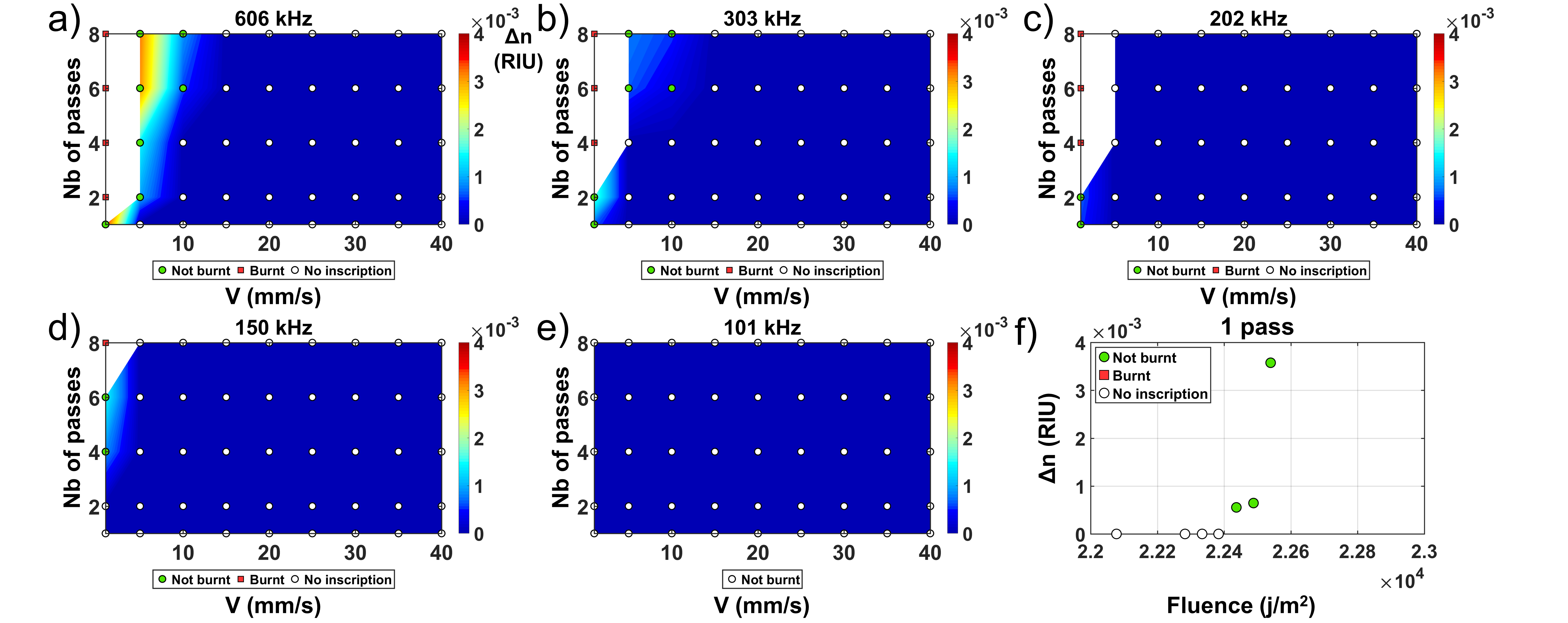


Supplementary Fig. S6: a), b), c), d) and e) Fs laser induced refractive index change in PDMS-germaniun-ATEG for various writing speed and numbers of passes at a given repetition rate. f) Refractive index change evolution relatively to the fluence for one pass by varying incrementally the writing speed and/or the repetition rate.

The maximum refractive index difference achieved was of 3.6 x 10 ^-3^ which is higher than the 2.2 x 10^-3^ significant RI change is localize around a precise set of parameters which is not ideal. We can observe on supplementary Fig. S6, that only few recipes did produce an RI change.

Germanium-Acrylate (MACMTG)

Germanium-Acrylate was another promising germans derivative susceptible of increasing the photosensitivity of PDMS. A concentration of 3 % mass was mixed with the PDMS before curing. The RI evolution regarding the recipe used in this study is presented at supplementary Fig. S7.


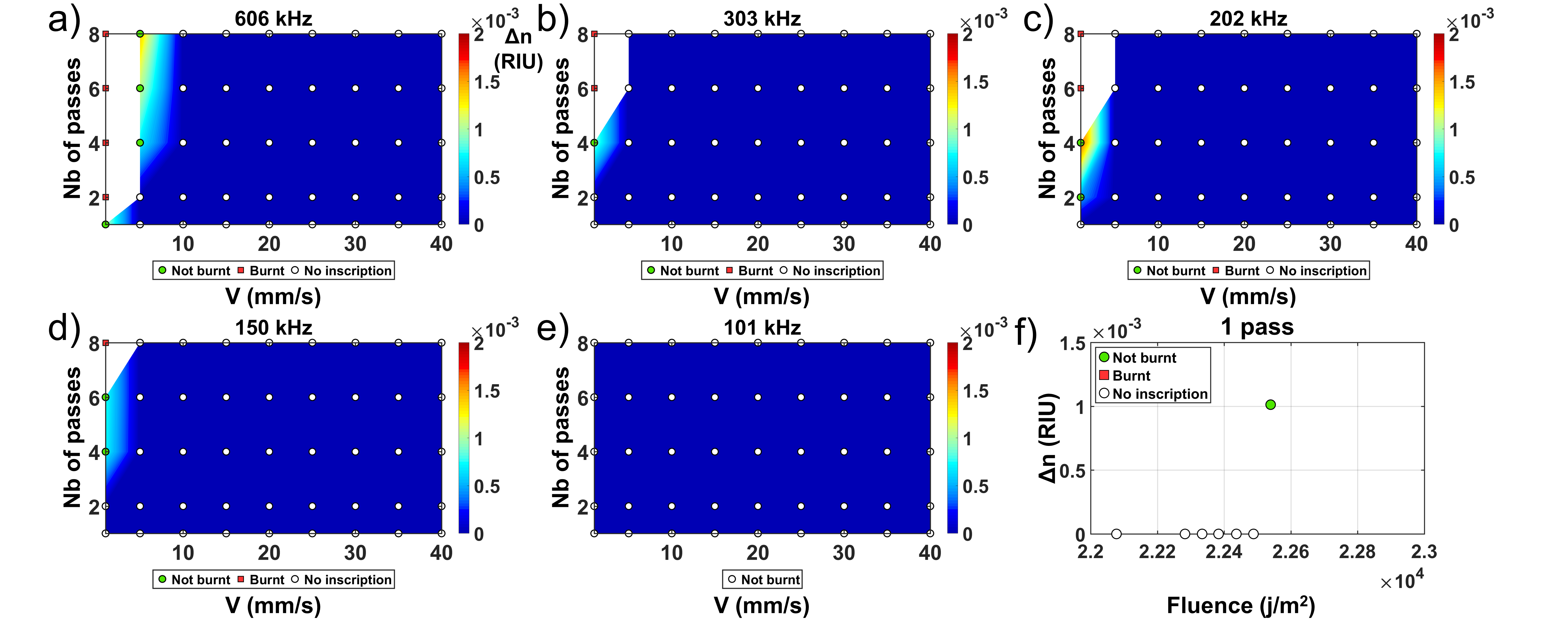


Supplementary Fig. S7: a), b), c), d) and e) Fs laser induced refractive index change in PDMS-germaniun-acrylite for various writing speed and numbers of passes at a given repetition rate. f) Refractive index change evolution relatively to the fluence for one pass by varying incrementally the writing speed and/or the repetition rate.

The maximum RI achieved was of 1.4 x 10^-3^ which is comparable to the pristine PDMS itself. The writing area is well localised around a narrow set of parameters rendering this chemistry les photosensitive relatively to the other compound.

Zirconium isopropoxide

Incorporation of this Zirconium into PDMS results of agglomeration resulting in a whiteish material. Removal of curing agent (Part A of Sylgard-184) solves this problem; therefore, the zirconium also acts as a curing agent suggesting a proper inclusion of this material into PDMS chain. In this study, we used a mixing ratio of 0.11 % weight of Zirconium and we did measure the RI change induced by different recipe as it is shown in supplementary Fig. S8.


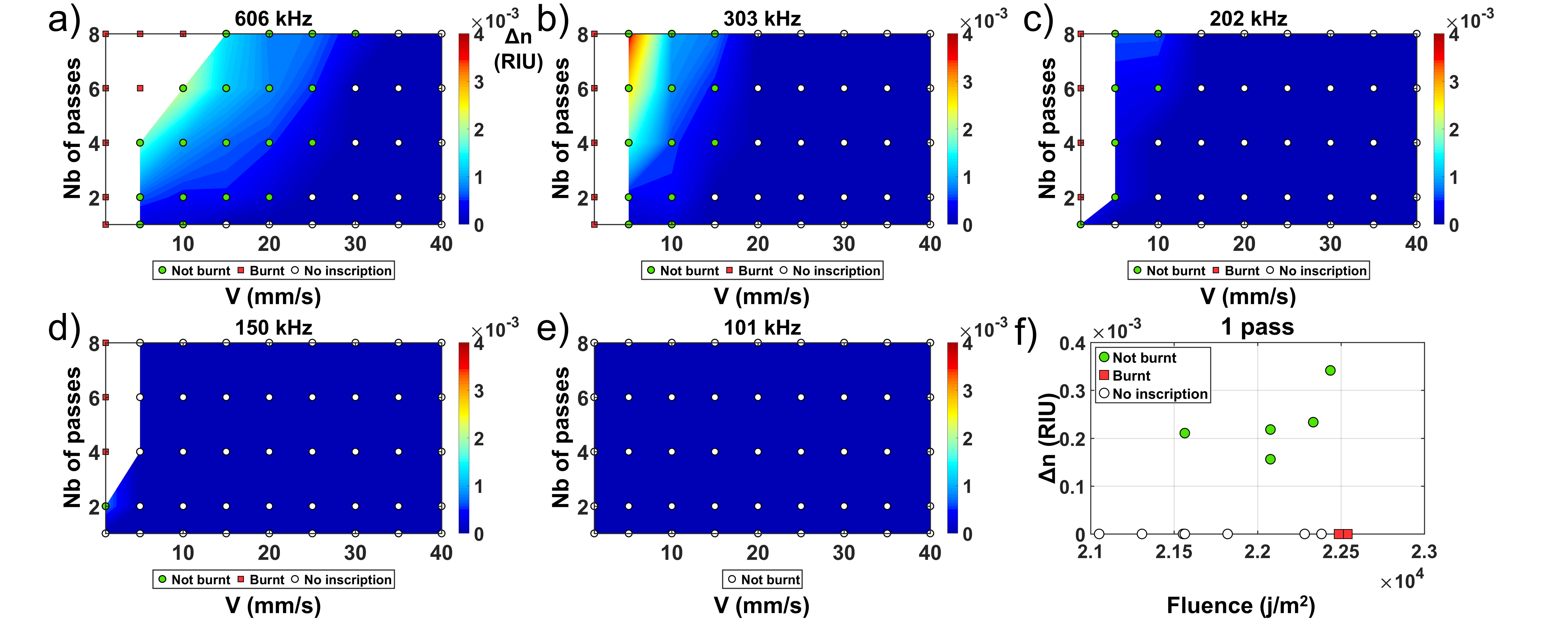


Supplementary Fig. S8: a), b), c), d) and e) Fs laser induced refractive index change in PDMS-zirconium for various writing speed and numbers of passes at a given repetition rate. f) Refractive index change evolution relatively to the fluence for one pass by varying incrementally the writing speed and/or the repetition rate.

The maximum RI measured was of 3.7 x 10^-3^ which is 3 times higher than the pristine PDMS. If we look at the fluence, one can observe that we have detected and undetected event in the same area of fluence. Here again, the detected events are systematically the one at lower writing speed as the undetected event are at higher speed but with an even higher variability than the BP case. Regarding the single pass analysis, the maximum RI achieved is the same as the pristine PDMS.

Titanium oxide derivatives

Concentration of 0.1 % was incorporated without any sign of aggregate but used of higher concentration (1%) result of aggregation rendering the sample unsuits for laser writing. The RI change relatively to our different parameters is shown on supplementary Fig. S9.


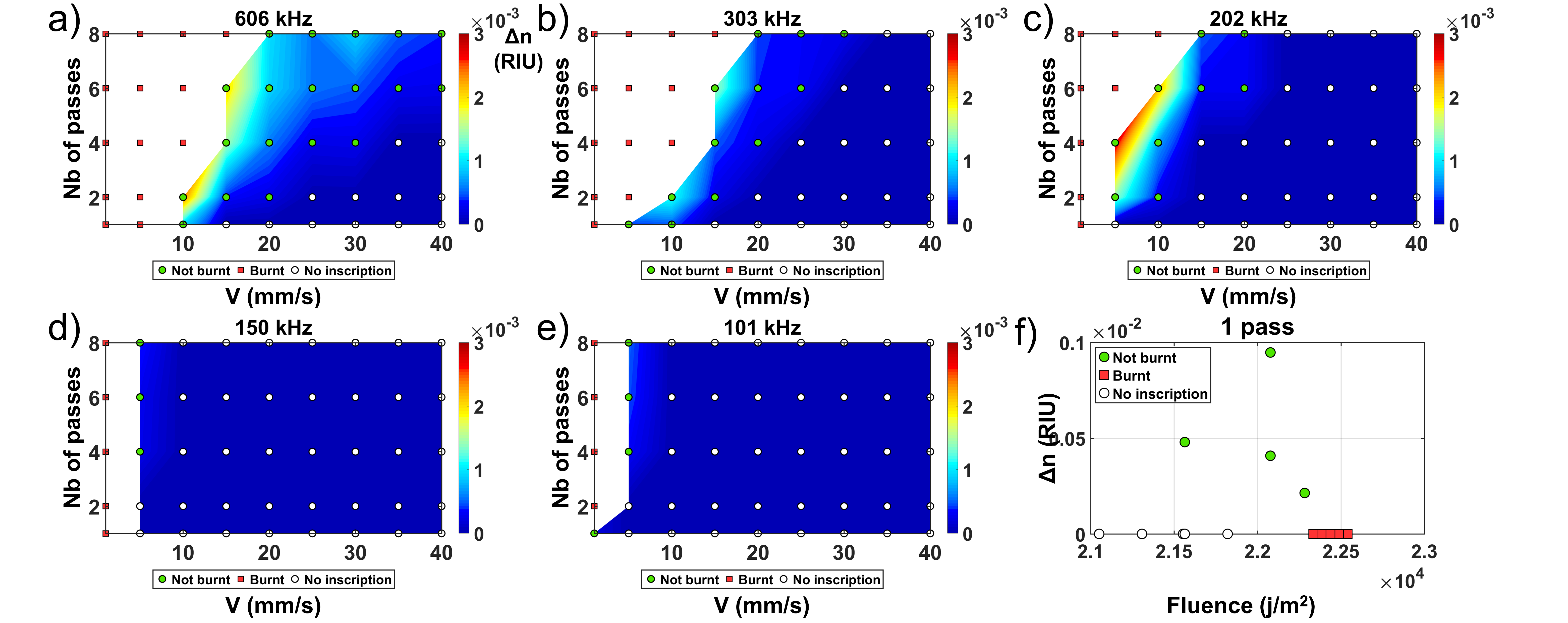


Supplementary Fig. S9: a), b), c), d) and e) Fs laser induced refractive index change in PDMS-TiO_2_ for various writing speed and numbers of passes at a given repetition rate. f) Refractive index change evolution relatively to the fluence for one pass by varying incrementally the writing speed and/or the repetition rate.

The maximum RI measured was of 2.8 x 10^-3^ which is twice higher than the maximum of the pristine PDMS, but the WWA is much lower (0.005) which indicates that only a few sets of parameters yield results. Maximum RI change for a single inscription can reach up to 3 times higher compared to pristine PDMS. The fluence graph supplementary Fig. S9 (f) shows that there is an overlap between detected and undetected events at the same or higher fluence. Again, the detected event occurs at lower writing speed-repetition rate. This behavior seems as a resulting of the same subdynamics discussed in the Bp section. This behavior is also observed in Irgacure-1173 and Zirconium as shown in supplementary material.

Despite an increase of the maximum RI, this material shows a poor increase in photosensitivity. This could be due to the low concentration that we were able to incorporate without causing aggregate into the PDMS. Therefore, a functionalization of TiO_2_ particle to ensure well blending and incorporation is probably required which increases the complexity of using this agent relatively to the others to avoid cluster formation.

Irgacure-500

Full data set of the refractive index change for 4 %-wt of Irgacure-500.


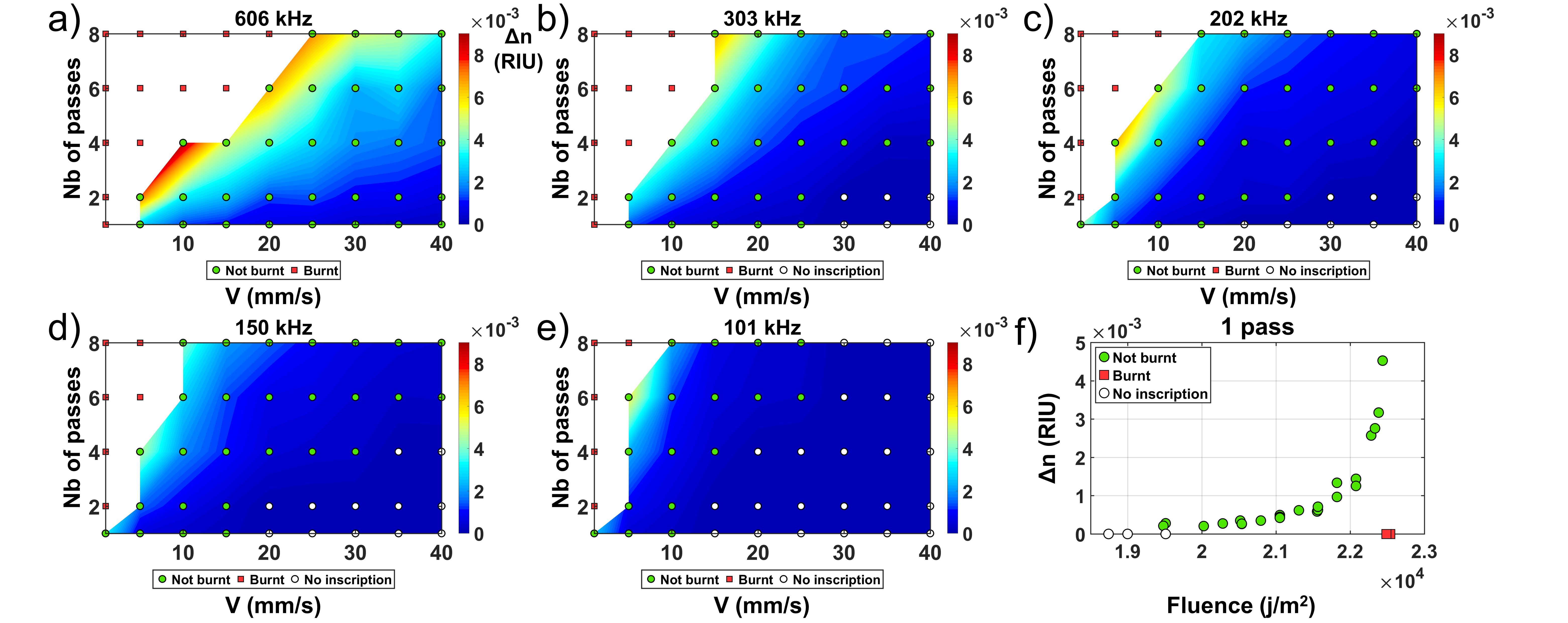


Supplementary Fig. S10: a), b), c), d) and e) Fs laser induced refractive index change in PDMS-TiO_2_ for various writing speed and numbers of passes at a given repetition rate. f) Refractive index change evolution relatively to the fluence for one pass by varying incrementally the writing speed and/or the repetition rate.

Measured RI changes for in Irgacure-500 samples for 50 inscriptions each separated by 100 μm under the same writing conditions.


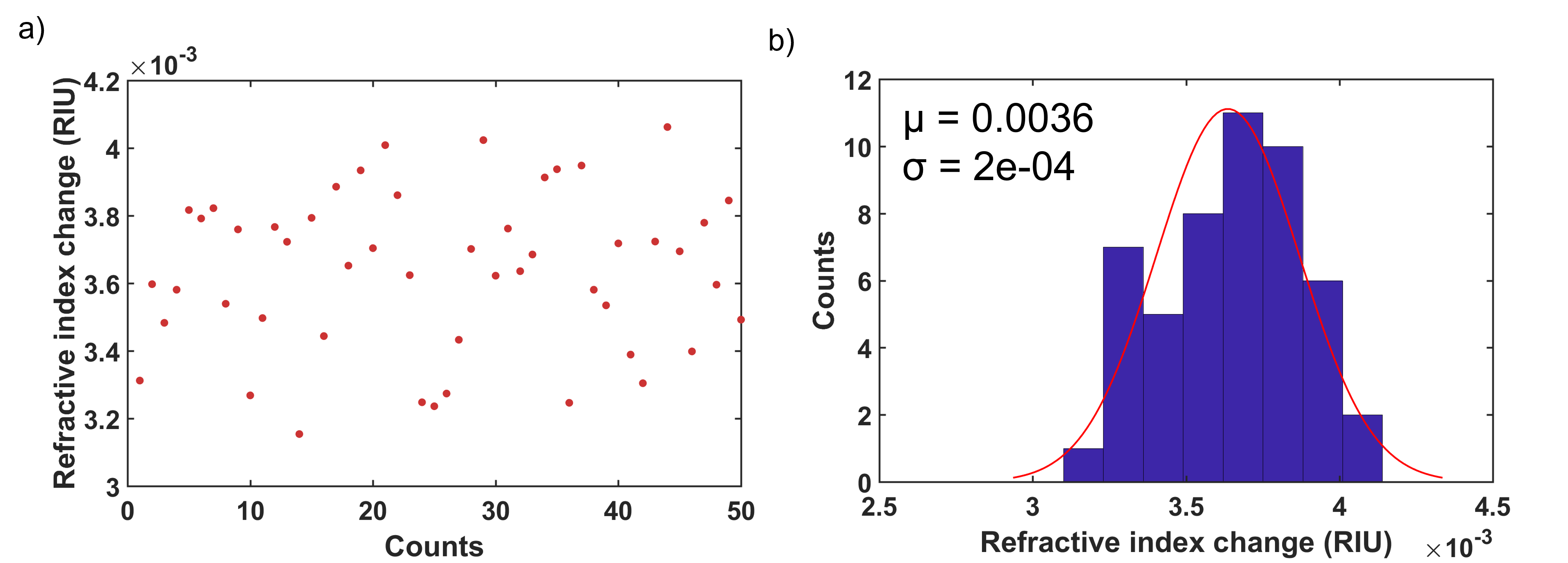
 Supplementary Fig. S11: a) Refractive index change of 50 different inscriptions written with the same parameters (4 passes, 4 mW, 606 kHz) in Irgacure-500 samples each separated by 100 μm and b) the Gaussian distribution generated to extract the mean value μ and σ the standard deviation (STD) from which the statistical uncertainty of the measurement can be calculated at a confidence level of 95%.
